# Adrenomedullin: Not Just Another Gastrointestinal Peptide

**DOI:** 10.3390/biom12020156

**Published:** 2022-01-18

**Authors:** Sonia Martínez-Herrero, Alfredo Martínez

**Affiliations:** Oncology Area, Center for Biomedical Research of La Rioja (CIBIR), C/Piqueras 98, 26006 Logroño, Spain; smherrero@riojasalud.es

**Keywords:** adrenomedullin, PAMP, microbiota, gastrointestinal peptides, intestinal physiology, intestinal pathology, inflammatory bowel disease

## Abstract

Adrenomedullin (AM) and proadrenomedullin N-terminal 20 peptide (PAMP) are two bioactive peptides derived from the same precursor with several biological functions including vasodilation, angiogenesis, or anti-inflammation, among others. AM and PAMP are widely expressed throughout the gastrointestinal (GI) tract where they behave as GI hormones, regulating numerous physiological processes such as gastric emptying, gastric acid release, insulin secretion, bowel movements, or intestinal barrier function. Furthermore, it has been recently demonstrated that AM/PAMP have an impact on gut microbiome composition, inhibiting the growth of bacteria related with disease and increasing the number of beneficial bacteria such as *Lactobacillus* or *Bifidobacterium*. Due to their wide functions in the GI tract, AM and PAMP are involved in several digestive pathologies such as peptic ulcer, diabetes, colon cancer, or inflammatory bowel disease (IBD). AM is a key protective factor in IBD onset and development, as it regulates cytokine production in the intestinal mucosa, improves vascular and lymphatic regeneration and function and mucosal epithelial repair, and promotes a beneficial gut microbiome composition. AM and PAMP are relevant GI hormones that can be targeted to develop novel therapeutic agents for IBD, other GI disorders, or microbiome-related pathologies.

## 1. Introduction

The digestive system is composed of the oral cavity, salivary glands, gastrointestinal (GI) tract, the liver, and the exocrine pancreas. The principal functions of the GI tract are to digest and absorb ingested nutrients and to excrete waste products of digestion [1]; but they are not limited to those. The GI tract is the key interface between ingested nutrients and the body, and it plays a critical role in regulating energy homeostasis [2]. Furthermore, recent findings have demonstrated that the events that take place in the gut during early life contribute to the programming, shaping, and tuning of GI tract physiology, the microbiome, and the immune system, having lifelong health consequences [3].

The GI tract can control and exert different actions in other organs thanks to multiple GI-derived signals including peptides/hormones, bile acids, and biomolecules [4]. In fact, the GI tract represents the largest endocrine organ in the body thanks to enteroendocrine cells (EECs) harbored all along the gut. EECs are the primary sensors of changes that take place in the GI tract, and they synthesize and release an array of peptides and hormones that act as autocrine, paracrine, or endocrine regulators of digestive function, glucose homeostasis, and energy balance, among other functions [5]. Over 100 different peptides with hormonal activity are produced and released by EECs as well as neurons distributed along the GI tract. GI peptides/hormones play an integral role in regulating the functions of the GI tract. In addition to their regulatory effects on secretion, absorption, digestion, and gut motility, they also have a fundamental role in gut–brain communication, particularly in the control of food intake and energy homeostasis [6]. Several GI-derived peptides are also synthesized within the brain, where they act as neuromodulators and/or neurotransmitters [4]. In this context, another important player has more recently emerged, the gut microbiota, whose interaction with GI hormones seems to have a key role in the effects of diets and improving the treatment of some intestinal disorders [7,8]. This adds a layer of complexity in terms of characterizing the physiologic role of GI-derived peptides.

Adrenomedullin (AM) and proadrenomedullin N-terminal 20 peptide (PAMP) are two small active hormones derived from the expression of a single gene (*Adm*) that is expressed throughout the GI tract, including the mucosal epithelium, glandular duct cells, neuroendocrine cells, and smooth muscle cells of the GI tract, between the oral cavity and the rectum [9,10,11,12]. These two peptides coexist in GI cells, where they regulate many physiological functions including vasodilation, angiogenesis, anti-inflammation, organ protection, and tissue repair. AM suppresses inflammatory cytokine production in the intestinal mucosa, improves vascular and lymphatic function, mucosal epithelial repair, and intestinal barrier function in animal models with intestinal inflammation [10,13,14,15]. Different research groups have demonstrated that AM has a protective role in many GI diseases, and its administration in rodents and humans ameliorates the severity of different gut pathologies such as gastric ulcer [16] or inflammatory bowel disease (IBD) [17,18]. Since AM is an endogenous bioactive peptide, it has low immunogenicity and is considered relatively safe, so it is expected that novel AM- and/or PAMP-derived treatments for GI pathologies may be developed in the coming years [19].

The purpose of the present review was to analyze the current status of the knowledge of AM and PAMP actions as gut-derived peptides, their relationships with some of the main GI disorders, including microbiota modulation, and their possible use as a novel therapeutic approach for gut pathologies.

## 2. Adrenomedullin and Proadrenomedullin N-Terminal 20 Peptide

AM was initially discovered in 1993 by Kitamura et al. [20] when it was isolated from a human pheochromocytoma. AM was initially studied as a peptide able to activate platelet adenylate cyclase and exert a long-lasting hypotensive effect [20], but it was later found to promote angiogenesis, organ protection, and anti-inflammatory immune activity [9].

### 2.1. Adrenomedullin and PAMP Biosynthesis and Structure

AM and PAMP are both coded by the *Adm* gene located in human chromosome 11p15.4 and in mouse chromosome 7. This gene contains four exons and three introns, with TATA, CAAT, and GC boxes in the 5’-flanking region [21]. The *Adm* gene codes for a large precursor, preproAM, of 185 amino acids. PreproAM is converted into proAM after the cleaving of the 21-residue signaling peptide. The enzymatic processing of proAM generates both mature AM (amino acids 95–146 of preproAM) and PAMP (amino acids 22–41 of preproAM) [22]. An interesting fact is that although both AM and PAMP are produced by the same prohormone precursor, their expression may not coincide in the same cells due to the existence of an alternative splicing mechanism [23].

Human AM is composed of 52 amino acids and has a ring structure consisting of six amino acids and a C-terminal amide structure. Both structural features are essential for its biological activity. AM shares structural similarities with calcitonin gene-related peptide (CGRP), amylin, and intermedin (also known as AM2) [22,24,25]. Another common structural characteristic of the members of the CGRP family is the presence of a central alpha helix. In the case of AM, approximately one-third of its total length is occupied by this central helical region, which seems to be required for binding to specific receptors and, thus, for exerting physiological functions [26]. PAMP is also constituted by an alpha helix, which is important not only for receptor recognition but also for some of its actions such as its antimicrobial activity [27,28].

AM is widely expressed throughout the blood vessels, heart, lungs, kidneys, central nervous system, and GI tract, among others, and is highly concentrated in the adrenal medulla. PAMP has a shorter antihypertensive activity than AM and cooperatively regulates blood circulation with AM [23]. 

AM synthesis and release are mostly regulated by oxidative stress, hypoxia, and inflammation-related molecules such as lipopolysaccharide and pro-inflammatory cytokines, including TNFα and IL-6, which increase AM’s secretion rate [9,29]. 

### 2.2. Adrenomedullin and PAMP Receptors

The AM receptor consists of a complex of a seven transmembrane domain protein (calcitonin receptor-like receptor (CLR)) and a single transmembrane domain protein (receptor activity modifying protein (RAMP)). After one of the RAMPs bind to CLR in the endoplasmatic reticulum, the resulting complex is transported to the plasma membrane [30].

There are three different RAMP isoforms in the human genome: RAMP1, RAMP2, and RAMP3. The complex formed by the union of CLR and RAMP1 functions as a receptor for the CGRP peptide [30]. The CLR molecules that bind to RAMP2 or RAMP3 isoforms are core-glycosylated, and these are the complexes that work as AM receptors (AMR). CLR/RAMP2 is known as AMR_1_, whereas CLR/RAMP3 is called AMR_2_ [31]. It has been established that amino acid 74 in RAMP2 and RAMP3 is critical for their affinity for AM, while amino acid 93 in RAMP1 is mainly responsible for its affinity for CGRP [31].

RAMP2 is more abundantly expressed under physiological conditions. However, the balance between the expression of RAMP2 and RAMP3 in a particular cell type can change, determining the degree of response to AM [32]. Apparently, the elevation in RAMP3 expression occurs as a mechanism to decrease AM’s responsiveness in those situations in which AM levels are most elevated such as in pregnancy, sepsis, or heart failure [32].

Specific binding sites for AM have been described in almost all tissues and cell types, including heart, lung, liver, spleen, skeletal muscle, kidney, GI tract, brain, or spinal cord, among others [12], providing the anatomical basis for the involvement of AM in the physiology of all those organs. 

PAMP differs in size and sequence from AM; thus, it has no affinity to the AMR complex CLR/RAMPs. Instead, the Mas-related G protein-coupled receptor member X2 was proposed as the receptor for PAMP as well as for its endogenously processed form, PAMP-12 (consisting of amino acids 9–20 of the PAMP’s mature form) [33]. Some publications have shown that the cytoskeleton can also function as a sort of intracellular receptor of PAMP (34). Physiological experiments show that PAMP contributes to microtubule fluidity and increases kinesin speed [34].

Recent studies have proposed that atypical chemokine receptors (ACKRs) can also act as AM/PAMP receptors [35]. ACKRs are vital regulators of the spatiotemporal distribution of chemokines [36]. ACKR3, formerly named CXCR7, is expressed ubiquitously but is most abundantly present in different brain regions, adrenal glands, lymphatic and blood vasculature, heart, and various subsets of immune cells [37]. AM seems to be the only member of the CGRP peptide superfamily that moderately activates ACKR3 [35]. Remarkably, PAMP has an activity toward ACKR3 that is comparable to AM. Furthermore, its truncated analog, PAMP-12, shows a greater potency toward ACKR3 than AM [35].

### 2.3. Main Physiological Effects of Adrenomedullin and PAMP

AM/PAMP play a main role during mammalian embryonic development. Both peptides can be detected in the uterus, the placenta, and several fetal tissues [38]. Furthermore, AM is locally produced in the trophoblast binucleate cells of the bovine placenta, where it may play a critical role regulating placenta vascularization during pregnancy, especially during the transition of late gestational period [39]. The generation of different knockout (KO) models for the *Adm* gene support the idea that AM is intimately related with pregnancy and embryonic development. In KO mouse models where AM and PAMP synthesis are suppressed, the null phenotype is embryonically lethal [40,41]. The same results were observed when AM receptors are suppressed; CLR^−/−^ and RAMP2^−/−^ embryos die in utero at mid-gestation due to severe deformation, vascular fragility, severe edema, and hemorrhage [42,43]. Surprisingly, no survival problems were observed when the expression of RAMP3 was totally suppressed [44]. 

To circumvent the problem of embryo lethality, tissue-specific conditional KO models were generated to study the actions exerted by AM/PAMP in adult organisms [45]. 

Systemic administration of AM reduces arterial blood pressure, decreases peripheral vascular resistance, and increases heart rate and cardiac output [46]. Although AM acts as a vasodilator when administered peripherally, it acts as a vasoconstrictor when it is injected into the brain, probably acting through vascular nerve terminals [47]. AM and PAMP are also well known for being potent angiogenic peptides [48], they are necessary for maintaining the integrity of the microvasculature [49] and promoting a faster healing of epithelial wounds [50,51]. 

Acting directly in the kidneys and through the hypothalamic–pituitary axis, AM is able to control renal function and regulate body fluid volume [52,53]. Interestingly, PAMP is expressed in the juxtaglomerular complex and co-secreted with renin, so this small peptide may be also involved in fluid volume control [54].

AM also regulates the secretion of other hormones. Maybe the best described to date is the regulatory effect of AM on blood glycemic levels via regulating insulin secretion. AM has been shown to reduce insulin secretion, and the use of a blocking monoclonal antibody against AM was able to increase the insulin secretion rate five-fold [55]. AM also regulates ghrelin secretion in the mouse stomach through an indirect mechanism mediated by plasmatic glucose levels [56], as it has been shown that low glucose induces ghrelin secretion from the stomach [57]. 

AM and its receptors are widely and abundantly expressed in the central nervous system [58]. This small hormone is an important neuroprotective agent against ischemic damage [59], increases preganglionic sympathetic discharges [60], and regulates some properties of the blood–brain barrier [61]. In addition, it has been described that AM may be able to regulate some behavioral responses such as stress and nociception [62,63].

Recently, a total inducible KO model for AM using Cre/LoxP technology combined with an optimized form of reverse tetracycline-controlled transactivator has been generated [56]. This new model has allowed the deep characterization of some of the already described actions for AM, and has led to the discovery of new ones, including its activity in bone remodeling [56] or the regulation of gut microbiota [14], which will be further discussed in this review.

## 3. Adrenomedullin/PAMP’s Roles in the Digestive System under Physiological Conditions

**Figure 1:**
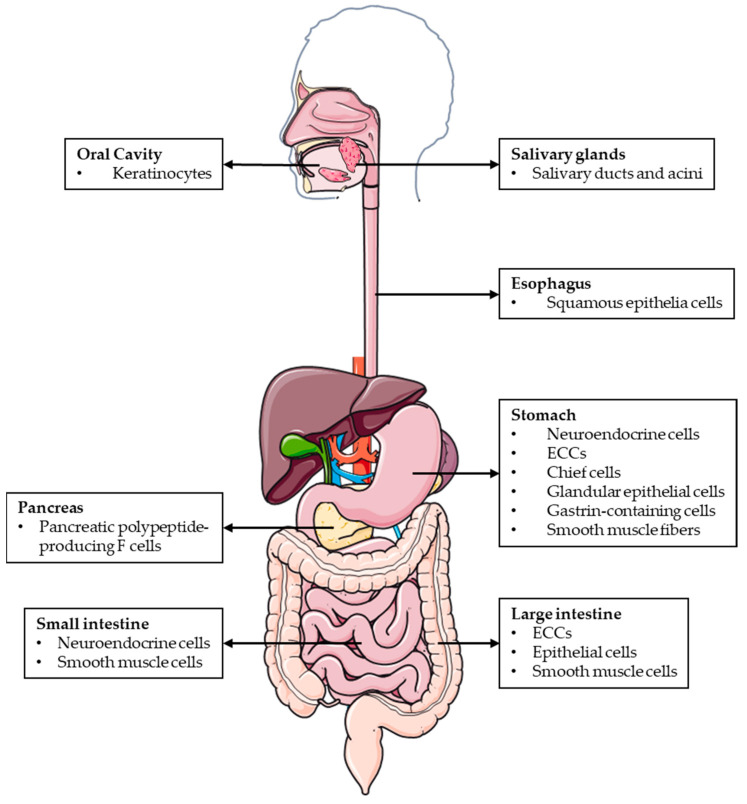
Schematic representation of the different locations where AM and PAMP are expressed throughout the GI tract.

AM and PAMP are widely expressed throughout the mucosal epithelium, glandular duct cells, neuroendocrine cells, and smooth muscle cells of the GI tract, between the oral cavity and the rectum [10] (Figure 1). The wide distribution of AM and PAMP in the GI tract suggests that both peptides regulate many digestive functions under physiological conditions.

### 3.1. Adrenomedullin Actions in the Oral Cavity

AM can be detected in the saliva, and it is secreted from oral keratinocytes and salivary glands [11,64]. Salivary AM seems to increase oral keratinocyte growth and to inhibit specific bacterial growth in the mouth in a dose-dependent manner [64]. Human oral keratinocytes resist bacterial infection, in part, by producing a broad-spectrum of antimicrobial peptides, including AM, to provide a robust response against pathogens [65]. Furthermore, microarray-profiling studies of dental caries revealed that the *Adm* gene is upregulated in pulp disease [66], probably as a defensive mechanism. 

### 3.2. Role of Adrenomedullin in Stomach Physiology

The expression sites of AM and its receptors in the stomach (especially abundant in the ECCs and chief cells of the gastric fundus and in the base of the glandular epithelia in the pyloric mucosa [67,68,69]) suggest that AM may play a role in regulating gastric functions in a paracrine manner. 

AM was first described as a potent inhibitor of basal gastric acid secretion in 1997 [70]. Further studies confirmed that AM was extremely potent in inhibiting basal and bombesin-, histamine-, and 2-deoxy D-glucose-stimulated gastric acid secretion [71]. Further, ex vivo studies suggested that AM acts via intramural fundic neurons stimulating somatostatin and, thus, inhibiting histamine and acid secretion in the stomach [72]. 

In addition to its actions on gastric acid release, AM is able to reduce total gastric juice volume and raise stomach pH values [73]. It has vasomotor effect on the gastric mucosa exerting a tight control of vascular function in physiological circumstances [74]. 

AM also inhibits gastric emptying in a dose-dependent manner [75]. This effect on gastric emptying may be related to a direct action of this peptide on smooth muscle cells, an effect that has also been linked to the synthesis of prostaglandins [76,77].

### 3.3. Adrenomedullin and PAMP Actions in the Intestine

AM expression in the intestine seems to be species specific. In the rat, half of the AM-like immunoreactivity is detected in the colon [69,78], while the ileum expresses the highest levels of preproAM among the small intestine [79]. AM immunostained cells can be localized in all intestine layers, intestinal nerves [80], and smooth muscle cells [81]. However, the major form of AM immunoreactivity in the colon corresponds with ECCs [78]. In the porcine GI tract, duodenal AM levels are about four to fourteen times higher than in other GI tissues, being more abundant in the mucosa and submucosa [82]. In human colonic mucosa, AM and PAMP immunostained cells are observed at a higher concentration in the apical region of the epithelium [11].

The fact that GI visceral smooth muscle cells express AM [69] suggests that AM may be able to regulate the contractile responses in the gut. This was proven in 2004, when AM elicited relaxation of the rat ileum in a concentration-dependent manner, acting through β_3_-adrenoreceptors [83]. Furthermore, intravenously injected AM disrupts phase 3 of the migrating motor complex (the cyclic motility pattern exhibited by the small intestine during fasting) in rat jejunum [79]. The migrating motor complex is regulated by a complex neuronal mechanism [84]; thus, AM control of the small-intestinal motility is probably achieved by acting as a neurotransmitter. Moreover, AM is able to regulate colonic bowel movements [81], causing a dose-dependent persistent relaxation of the rat colonic smooth muscle by elevating cAMP levels [81]. 

AM and PAMP can regulate sugar absorption by the enterocytes [85]. PAMP enhances sugar uptake, while AM inhibits the absorption of α-methylglucoside. Both peptides regulate sugar absorption via the recruitment of the sodium glucose co-transporter-1 (SGLT1) to the apical membrane [85]. AM also actively regulates water and ion absorption and transport in the colon [81]. This has two important effects on ion transport: it decreases sodium absorption and enhances chloride secretion [81]. By altering ion flow, AM also modulates water absorption in the colon.

### 3.4. Role of Adrenomedullin in Pancreatic Physiology

In the pancreas, AM expression appears early in embryonic development. Specifically, AM immunoreactivity appears at day 11.5 of embryonic development in the rat [86]. At some point during development, all pancreatic cell types express AM, but this evolves towards the adult pattern where AM is only express by the pancreatic polypeptide-producing F cells on the periphery of pancreatic islets of Langerhans [87].

The early appearance of AM during embryonic development suggests an active role in the growth and morphogenesis of the organ [86]. However, the best known action of AM in the pancreas is the inhibition of insulin secretion, thus modulating blood glucose levels [87]. AM inhibits insulin secretion in a dose-dependent manner, increasing circulating glucose levels at the same time, and a monoclonal antibody against AM is able to increase insulin release five-fold [87]. The inhibition of glucose-induced insulin secretion by AM was restored in the pancreatic β cells pretreating the cells with pertussis toxin, suggesting that this effect could be mediated by G proteins [88] and an elevation of cAMP [89].

Recent studies have provided evidence that AM/PAMP are involved in the regulation of ghrelin levels [56]. Ghrelin levels in the blood of total KO mice were significantly higher than in the WT littermates [56]. This may be a consequence of AM regulation of insulin secretion in the pancreas. Animals lacking AM/PAMP have lower fasting basal glucose levels than WT animals, and these differences are maintained through a glucose tolerance test [56]. It has been shown that low glucose induces ghrelin secretion from the stomach [57], so ghrelin regulation by AM/PAMP may be achieved through indirect mechanisms involving glycemic levels. 

AM is able to inhibit amylase secretion too, probably acting through a GTP-binding protein and reducing the calcium sensitivity of the exocytotic machinery of the pancreatic acini [90], however, this mechanism is not yet fully understood. 

### 3.5. Adrenomedullin/PAMP’s Impact on Microbiota Composition

Gut microbiota has emerged as a main regulator for many GI functions as well as a major player for maintaining a healthy GI function [91,92]; thus, any hormone/peptide able to regulate microbiota composition may have an impact on GI physiology. 

AM and PAMP are found in mostly all epithelial surfaces and body secretions including saliva, sweat, milk, and urine [22,93]; this suggests that both of them may play a role as antimicrobial peptides, contributing to host defense [94]. This was further confirmed by different studies using in vitro, in vivo, or clinical data. 

Plasmatic and GI AM levels increase in many infectious diseases [80,94,95], especially in sepsis [96], suggesting a protective role for this hormone.

The antimicrobial activity of AM and PAMP was demonstrated in 1996 [97] for the first time. These two peptides were able to suppress the growth of Gram-positive and Gram-negative bacteria in a concentration- and time-dependent manner [98,99,100]. 

AM shares many properties with other cationic antimicrobial peptides, including human β-defensin-2, and therefore may share a similar antimicrobial mechanism of action [94]. Ultrastructural studies have demonstrated that AM treatment causes a cell-wall disruption in *E. coli* and an abnormal septum formation in *S. aureus* [101]. This antimicrobial activity seems to be related with the carboxy terminal fragment of AM [101].

In the case of PAMP, a conformational analysis revealed that its structure is compatible with a pore-forming mechanism [27]. Antimicrobial analysis using radial diffusion and outer membrane permeability assays showed that free-acid and mature PAMP are very efficient in increasing outer membrane permeability in *E. coli*, in a similar way to polymyxin B [28] thus confirming the suspected antimicrobial mechanism of action for PAMP.

The inducible total KO model for AM and PAMP has allowed the confirmation of the effects of eliminating the *Adm* gene on gut microbiota in physiological conditions [14]. Abrogation of the *Adm* gene in the whole body caused significant changes in colonic microbiota: higher proportion of the *Proteobacteria* class and the *Coriobacteriales* order, and other families and genera was observed in KO feces. Meanwhile, these mice had a lower proportion of beneficial bacteria such as *Lactobacillus gasseri* and *Bifidobacterium choerinum* [14]. All together, these data point to a beneficial effect of AM/PAMP on GI tract health. 

## 4. Pathophysiological Involvement of Adrenomedullin/PAMP in the Most Relevant Diseases of the Digestive System

The pathophysiological function of AM in GI diseases has been reported in many studies on the stomach, small and large intestine, and pancreas.

### 4.1. Adrenomedullin and Peptic Ulcer Disease

Peptic ulcer disease (PUD) is a very common condition worldwide. Its incidence varies from country to country, being the highest in Spain, with an annual incidence of 141.9 per 100,000 habitants in 2018, and the lowest in the UK, with 23.9 cases per 100,000 habitants [102]. This disease represents one of the major burdens for healthcare systems in the 21st century [102].

AM gastroprotective action was demonstrated for the first time in 1998 when Kaneko et al. [103] demonstrated that AM exerts a protective action against ethanol-induced gastric lesions when injected peripherally. However, intravenous administration of AM seems to have no effect on the development of gastric mucosal lesions, suggesting that AM-mediated protective action should involve the sympathetic nerve system [16]. 

AM is able to regulate stomach pH through its anti-secretory activity involving somatostatin [104], thus providing another mechanism to explain its gastroprotective activity.

Furthermore, peptic ulcers are accompanied by marked circulatory disturbances, leading to perivascular edema and microhemorrhages [105]. AM is able to increase blood flow in the gastric mucosa, improving arterial blood flow, which has been proven to be particularly beneficial for mucosa healing [74].

The regeneration of the gastric epithelium is another endogenous mechanism against superficial injury in the stomach. In vitro studies have demonstrated that AM promotes and accelerates epithelial restitution and enhances the recovery of the mucosal integrity after mild damage [68].

It has also been demonstrated that AM is overexpressed in different cell types of the stomach during the healing and scarring stages of gastric ulcer [106]. This observation supports the hypothesis that AM may be useful for tissue repair.

Taken together, all these studies suggest that AM could be considered as a new therapeutic target in the development of new drugs against peptic ulcer disease. 

### 4.2. Adrenomedullin and Intestinal Ischemia

Acute mesenteric ischemia (AMI) is a common emergency that affects 5.5% of the population annually and with a very high mortality rate (60–80%) [107,108]. This condition has a difficult diagnosis and treatment [109], which highlights the need to develop a novel approach to treat this pathology. 

Emerging studies are providing evidence that co-administration of AM and AM binding protein 1 could prevent injury after the ischemic episode [110,111]. These findings are supported by many other studies where AM beneficial action against ischemia/reperfusion injury has been reported [59,112,113,114]. AM treatment downregulates the expression of several pro-inflammatory cytokines, preserves gut barrier function, improves perfusion, and improves survival rate [110,111].

### 4.3. Adrenomedullin and Diabetes

AM emerged as a fundamental peptide to maintain insulin homeostasis and normoglycemia. The inhibitory effect of AM on insulin release suggests that this peptide may be associated with diabetes mellitus. Clinical data show that AM is elevated in some groups of both type I and II diabetic patients [115]. Furthermore, AM plasmatic levels are further increased in those patients with extra-pancreatic complications [115]. 

Increases in plasma AM in type 1 diabetes seem to be a consequence of the disease, because it is increased only in patients with microangiopathy and with diabetic nephropathy [115,116]. In type 2 diabetes, it is not clear whether AM elevation is a consequence or a causal agent of the disease, but it is undeniable that the levels of circulating AM are elevated in these patients [117,118]. In addition, AM has emerged as a possible biomarker for early diagnosis of pancreatic cancer-induced diabetes [119].

### 4.4. Adrenomedullin and Cancer in the Digestive System

Perhaps the disease that is more clearly influenced by AM expression is cancer [119]. The involvement of AM in tumor progression is becoming more evident every day. Most studies support the idea of AM as a survival factor for tumor cells; a factor that can be produced either by the tumor itself [120,121] or by the stromal cells surrounding the tumor [122]. This peptide is a good investment for the tumor cell, because it is involved in tumor initiation and progression, by promoting cell proliferation, angiogenesis, change of phenotype, inhibition of apoptosis, and escape from immune surveillance [123].

In the digestive system, there is significant evidence for the association of the expression of AM and its receptors with cancer. AM and CLR mRNA levels were higher in patients with insulinoma and in pancreatic adenocarcinoma tissues compared to normal pancreatic tissues [124,125], suggesting that AM was directly produced by tumor cells. Although the expression of AM, CLR, RAMP1, and RAMP2 mRNA has been reported in several pancreatic cancer cells, RAMP3 mRNA expression could only be found in one of five cancer cell lines studied [124]. These observations indicate that RAMP1/2 but not RAMP3 are the main co-receptors for CLR in pancreatic adenocarcinoma [124]. Hypoxia seems to be the major trigger of AM overexpression [124,126]. Recent studies have also identified AM as a potential mediator of diabetes in patients with pancreatic cancer [126,127]. These studies point out that AM is most likely acting as a mediator of new-onset and concomitant weight loss in these patients. 

Furthermore, the expression levels of AM and its receptors are inordinately elevated in gastric cancer as compared to the adjacent non-tumor gastric tissues [128], and increased AM expression results in the proliferation of tumor cells, tumor invasion, and metastasis.

Human colon carcinoma cells (HT-29, HCT116, DLD1, and SW480) express AM, CLR, RAMP2, and RAMP3, and in all of them, the expression of AM increased under hypoxic conditions [121,122,129]. Addition of synthetic AM to tumor cells in culture stimulated cell proliferation and invasion, which could be reversed by co-incubation with an AM antibody or an AM antagonist [129]. Furthermore, AM antibody treatment significantly reduced the growth of HT-29 tumor xenografts in mice [129]. These data seem to correlate well with clinical data where AM has been described as an independent prognostic factor for colorectal cancer [121]. Tissue microarray analysis of human colorectal tumors revealed that the expression levels of AM, CLR, RAMP2, and RAMP3 were higher in lymph nodes and distant metastasis when compared with primary tumors [129]. 

Although several studies have provided evidence that AM is involved in tumor initiation and progression in the colon, it has been reported that the use of a small molecule (SM) that positively enhances AM activity has a protective role during the progression phase of colon cancer associated to colitis [130]. This beneficial action is due to the fact of AM’s protective action against chronic inflammation. These data suggest that AM’s role in cancer initiation and progression may be different depending on tumor’s origin; thus, future studies should address whether AM is also protective or not depending on the specific genesis of colon cancer. 

### 4.5. Adrenomedullin/PAMP and Inflammatory Bowel Disease

The term IBD comprises Crohn’s disease (CD) and ulcerative colitis (UC), both are chronic pathologies characterized by inflammation of the GI tract [131]. UC causes swelling and ulcers in the colon, while CD can cause inflammation in any part of the GIT, from the mouth to the anus. The pathophysiology of IBD is unknown but is believed to be influenced by genetic and environmental factors. When these factors are coupled to alterations in gut microbiota, they lead to a deregulation of the immune response [132,133]. Their exact etiologies remain unknown, but clinical studies suggest that interplay between genetic factors and enteric bacteria are crucial for disease development, owing to abnormal host responses directed against the commensal microbiota [131]. In normal gut, intestinal epithelial cells maintain a beneficial link with the microorganisms in the intestinal flora through toll-like receptors (TLRs) that mediate signaling to maintain epithelial cell integrity and tight junctions [134,135]. Stimulation from commensal bacteria is finite and should not trigger an excessive inflammatory response [135]. However, any alteration in the population of luminal bacteria may influence TLR signaling, paving the way to a dysregulated inflammatory response, thus being a common denominator of IBD and colorectal cancer [134].

AM is emerging as a novel and promising therapy for immunological disorders [136,137], including autoimmune digestive pathologies, such as IBD, due to several local and systemic actions as described below.

#### 4.5.1. AM as an Anti-Inflammatory Factor

It has been demonstrated that AM inhibits the secretion of pro-inflammatory cytokines when it is released into the medium by peripheral blood monocytes when transforming into macrophages [22]. In addition to its regulatory role on immune cells, AM also decreases endothelial permeability, thus reducing the formation of inflammatory exudates [22].

Treatment of induced colitis with AM in animal models ameliorates the severity of the clinical symptoms and reduces the histological damage in the colon by modulating the inflammatory response. Intraperitoneal administration of AM has a potent anti-inflammatory effect, both at the local and systemic level. AM helps to maintain the Th1/Th2 balance, prevents neutrophil infiltration, and inhibits the release of several pro-inflammatory cytokines (IL-6, IL-10, TNF-α, and IFN-γ) in intestinal mucosa and mesenteric lymph node mononuclear cells [138,139,140]. It has been proposed that AM can exert these anti-inflammatory actions via the hypoxia-inducible factor (HIF): high levels of AM promote the synthesis of HIF-1α, an endogenous protective factor against inflammation in the mucosa [141]. Another mechanism recently proposed for AM anti-inflammatory actions is the STAT3–NFκB pathway: AM is able to inhibit NFκBp65 and STAT3 phosphorylation when administered subcutaneously [142].

Results from the inducible total KO model for *Adm* also demonstrated that endogenous AM is necessary to maintain a proper immune response against colonic damage [14,15]. Lack of AM/PAMP results in an abnormal immune response in the two principal chemical-induced models of colitis (i.e., DSS and TNBS) with an overexpression of pro-inflammatory cytokines (IL-1β, IL-6, and TNF-α) and a downregulation of anti-inflammatory cytokines (IL-10) [14,15].

#### 4.5.2. AM’s Role in Maintaining Intestinal Epithelial Barrier Integrity

AM has proven to be essential in the maintenance of intestinal epithelial barrier function, preventing increased permeability and hyper-activation of the intestinal epithelium, both of them hallmarks of IBD [139]. This protective effect of AM on intestinal epithelial barrier function may be mediated via suppression of inflammatory cytokines and downregulation of myosin light-chain phosphorylation, which is a key regulator of intestinal barrier function [143]. In addition to these data, it has also been proven that endogenous AM may have a direct effect on the expression of key proteins for the epithelial barrier function in the enterocytes, including junctional adhesion molecule A (JAM-A) and e-cadherin. Lack of AM/PAMP results in lower expression levels of JAM-A and e-cadherin, both of which are necessary to maintain the intestinal barrier after the colitis insult [15].

#### 4.5.3. AM Improves Mucosal Healing and Re-Epithelialization

AM’s role in promoting a faster healing and restitution of epithelial lesions in the skin and gastric tissue has already been described [50,68]. It has also been proven that AM administration ameliorates the symptoms of DSS-induced experimental colitis through acceleration of ulcer re-epithelialization and mucosal regeneration in the colon [144].

Furthermore, the expression of different regenerative biomarkers (i.e., Lgr5, Errb2, Egfr, and Wnt5a) was significantly lower in null AM animals after the colitis insult when compared with their WT littermates [15], suggesting that AM may be regulating and promoting the regeneration and renewal of colonic epithelium via direct actions on the enterocytes. 

#### 4.5.4. AM Actions on Enteric Vasculature

As a potent angiogenic factor [48], AM is necessary for maintaining microvasculature integrity in the mucosa [49]. Activation of the nitric oxide (NO) pathway by AM has a main role in the regulation of the cardiovascular system by regulating blood flow [145]. It has been described that AM protects and restores mesenteric vascular function in the colon by decreasing cyclooxygenase-2 expression (an enzyme that mediates inflammatory processes) in microvessels of rats with colitis and NO expression in colonic tissue but not at the systemic level [146]. Furthermore, a mouse model with a lymphatic-specific KO of the CLR gene of the AMR was recently developed [147]. It was found that these animals developed intestinal lymphangiectasia, suggesting that AM protective actions against intestinal inflammation may be mediated through its effects on lymphatic function.

#### 4.5.5. AM and PAMP Modulation of Intestinal Microbiome

As explained above, intestinal microbiota has emerged as a key factor in maintaining a healthy gut. Abnormalities in the function and/or composition of the enteric microbiome is associated with IBD onset and worst symptoms [132]. 

AM’s antimicrobial effects [148] could help fight the overgrowth of inflammation-related bacteria in the microbiota as has been described in IBD patients [134,135]. AM administration to mice subjected to DSS-induced colitis significantly reduced the number of anaerobic bacteria in the colon when compared to control mice [149]. Furthermore, mice lacking AM have a higher proportion of *Proteobacteria* and *Coriobacteriales* order (bacteria related with inflammatory outcomes [150]) and a lower proportion of beneficial bacteria such as *Lactobacillus* and *Bifidobacterium* [15]. These results suggest that the antimicrobial activity of AM may mechanistically contribute to the maintenance of a healthy microbiome, thus improving colitis outcome. However, AM may have another effect on microbiota, modulating how intestinal bacteria communicate with the enterocytes via TLRs [134,135]. AM depletion causes changes in colonic TLR4 gene expression, causing an overexpression of this receptor in the intestinal epithelium, allowing bacteria to overstimulate the enteric immune system, which might lead to a worse prognosis during colitis episodes [14].

## 5. From Animal Models to Patients: New Pharmacological Treatments for Gastrointestinal Disorders Based on Adrenomedullin

### 5.1. Adrenomedullin-Based Therapies for IBD

By significantly reducing the severity of inflammation and other histological damage, treatment with AM helps to ameliorate the overall rate of disease severity, to control weight loss, diarrhea, intestinal bleeding, and to increase survival rate of treated animals in murine colitis models. After the success achieved in animals, some scientific groups translated these results to IBD patients and administered, for the first time, AM as a treatment for patients with refractory UC [151,152]. The results strongly suggested that AM had potential as a new therapeutic agent for the treatment of UC; thus, intravenously administered AM reduced in all cases the disease severity index between five and twelve points on a twelve point scale and accelerated healing. In several patients, it maintained clinical remission for over one year, with no observed secondary effects beyond a slight decrease in blood pressure. 

In patients with refractory CD with secondary failure of infliximab, continuous intravenous infusion of AM resulted in marked improvement in clinical symptoms and mucosal healing of longitudinal ulcers of the colon as well as a re-elevation of infliximab levels in the blood [18]. 

One of the most important advantages of a therapeutic use of AM is that it is a hormone naturally produced by the body; thus, it has a high safety profile. Furthermore, exogenous administration of AM has already been tested in previous clinical trials and pilot studies with little to no side effects [153,154,155,156]. On the other hand, AM has a short half-life (less than 60 min) [22], thus making a prolonged intravenous administration necessary [19]. Although continuous intravenous infusion is acceptable for remission induction for patients already hospitalized, it is not convenient for other patients or remission therapy [19]. To circumvent the problem of the quite short half-life of native AM in blood, Nagata et al. [157] developed and validated a chemically modified human AM, using a polyethylene glycol conjugated form of AM. 

Other alternatives may be the use of small molecules (SMs) that selectively inhibit or enhance AM activity [130,158]. Several SMs that are able to act as pharmacological modulators of AM and PAMP have been described and they can be used to intervene in all physiological and pathological conditions where these peptides play a role [158]. IBD patients benefit from AM actions; thus, positive modulators of AM may be useful to treat this disease. Furthermore, in a colitis-associated colorectal cancer (CRC) model, the use of an SM that enhances AM activity was demonstrated to be effective at reducing the colitis symptoms and preventing the chronic inflammation that led to CRC initiation and progression [130].

### 5.2. Positive Effects of AM in PUD

Due to the high prevalence of *Helicobacter pylori* infection and widespread use of potent anti-inflammatory drugs, the incidence of PUD remains high worldwide [102]. Even though it is not a lethal disease in most patients, the quality of life for people suffering from PUD can be highly diminished due to the abdominal pain or night reflux, among other symptoms [102].

As already mentioned in Section 4.1 of this review, AM has the potential to be considered as a treatment against PUD. AM is able to regulate the stomach’s pH [104]; it increases blood flow in the gastric mucosa, improving arterial blood flow (needed for mucosa healing) [74]; promotes and accelerates epithelial restitution after mild damage [68]; and it has already been proven to heal colonic ulcers in IBD patients [149]. All these observations support the hypothesis that AM may be useful for gastric tissue repair. Furthermore, its antimicrobial action may be beneficial when PUD is due to *H. pylori* infection. 

However, a very recent study [159] revealed that AM expression was positively correlated with the degree of gastritis in both mice and humans affected by *H. pylori*-induced gastritis. Furthermore, blockade of AM using antibodies resulted in decreased inflammation within the gastric mucosa of *H. pylori*-infected mice. This indicates that AM’s previously described beneficial actions in the stomach are suppressed after *H. pylori* infection. The observed increased in AM levels after *H. pylori* infection [159] is an expected outcome, as AM levels are also increased in other infections and in sepsis [80,94,95,96]. AM increase occurs as a defensive mechanism against the bacteria. However, in the particular case of *H. pylori*-induced gastritis, AM may be inducing an abnormal and exaggerated inflammatory response that instead of resolving the infection is aggravating the inflammatory outcome. 

These data only highlight the need for more research to better understand the role of AM in PUD, depending on the origin of the disease, and if it could be used as a therapeutic target for this disease. The use of SMs that selectively can increase or decrease AM’s activity can be a good approach for PUD patients. The actions of these SMs in stomach’s physiology or pathology have not yet been tested, but they can open a new avenue of treatments for this pathology. 

### 5.3. AM as a CRC Treatment

As discussed before, AM’s role in CRC initiation and progression may be different depending on tumor’s origin.

In some cases, AM enhances tumor growth, vascularization, and migration [129,160]. The best therapeutical approach for these tumors may be the use of pharmacological modulators that block or reduce AM actions. Among the available options to reduce AM activity, the best studied so far in the context of CRC are the use of antibodies against the peptide or the receptor [129,161]. The use of anti-AM receptor antibodies significantly reduces HT-29 tumor cell proliferation in vitro, tumor growth, and angiogenesis in vivo [160]. Treatment of the tumors with anti-AM antibodies or AM_22–52_ (a fragment of the peptide that blocks the binding of the hormone to its receptor) also reduced tumor growth and vascularization and increased tumor apoptosis [129]. 

However, in the particular case of CRC derived from chronic inflammation, augmenting AM levels was the most effective strategy to reduce the number of tumors [130], probably due to the beneficial action of AM over inflammation, preventing the malignant transformation of the colonic epithelium. 

## 6. Conclusions

AM and PAMP are two biologically active peptides produced by the same gene with ubiquitous distribution and many physiological functions. With regard to the GI tract, they are expressed in different proportions depending on the organ and cell type. They are especially abundant in the neuroendocrine cells of the GI mucosa, and ECCs are reported to be the main cell type expressing the *Adm* gene. This distribution pattern is closely related to their physiological actions. Both peptides can act as GI hormones regulating different processes, including gastric acid secretion, gastric emptying, gut permeability, and intestinal motility, among others. They control all these GI physiological functions acting either directly on the GI tract or through specific receptors in the brain and through vagal cholinergic-dependent mechanisms. 

AM and PAMP are also related to several GI pathological conditions including peptic ulcer, intestinal ischemia, diabetes, GI cancer, or IBD, among others. Over the past years, a growing body of evidence has suggested that both AM and PAMP can have a protective role on those diseases, especially those involving the immune system and inflammatory processes. The role of AM in IBD is attracting a lot of interest and some clinical studies have already started. The beneficial AM actions in IBD pathology include anti-inflammatory activity, vasculature homeostasis regulation, intestinal epithelium repair, intestinal barrier function restoration, and modulation of the microbiome composition. 

Furthermore, results of clinical research on AM therapy have demonstrated that a significant efficacy can be achieved at relatively low doses, where the side effect of hypotension caused by AM is not a clinical problem. Although further validation of the effective doses, dosages, and administration methods of AM therapy for IBD patients is needed, AM is considered to be a promising therapeutic agent for IBD or other GI disorders.

## Data Availability

Not applicable.

## References

[1] Hajishafiee M., Bitarafan V., Feinle-Bisset C. (2019). Gastrointestinal Sensing of Meal-Related Signals in Humans, and Dysregulations in Eating-Related Disorders. Nutrients.

[2] Monteiro M.P., Batterham R.L. (2017). The Importance of the Gastrointestinal Tract in Controlling Food Intake and Regulating Energy Balance. Gastroenterology.

[3] Milani C., Duranti S., Bottacini F., Casey E., Turroni F., Mahony J., Belzer C., Palacio S.D., Montes S.A., Mancabelli L. (2017). The First Microbial Colonizers of the Human Gut: Composition, Activities, and Health Implications of the Infant Gut Microbiota. Microbiol. Mol. Biol. Rev..

[4] Dockray G.J. (2014). Gastrointestinal hormones and the dialogue between gut and brain. J. Physiol..

[5] Guo X., Lv J., Xi R. (2021). The specification and function of enteroendocrine cells in Drosophila and mammals: A comparative review. FEBS J..

[6] Dockray G.J. (2013). Enteroendocrine cell signalling via the vagus nerve. Curr. Opin. Pharmacol..

[7] Xu X., Chen R., Zhan G., Wang D., Tan X., Xu H. (2021). Enterochromaffin Cells: Sentinels to Gut Microbiota in Hyperalgesia?. Front. Cell Infect. Microbiol..

[8] Cussotto S., Sandhu K.V., Dinan T.G., Cryan J.F. (2018). The Neuroendocrinology of the Microbiota-Gut-Brain Axis: A Behavioural Perspective. Front. Neuroendocrinol..

[9] Hinson J.P., Kapas S., Smith D.M. (2000). Adrenomedullin, a multifunctional regulatory peptide. Endocr. Rev..

[10] Martinez-Herrero S., Martinez A. (2016). Adrenomedullin regulates intestinal physiology and pathophysiology. Domest. Anim. Endocrinol..

[11] Marutsuka K., Hatakeyama K., Sato Y., Yamashita A., Sumiyoshi A., Asada Y. (2003). Immunohistological localization and possible functions of adrenomedullin. Hypertens Res..

[12] Lopez J., Martinez A. (2002). Cell and molecular biology of the multifunctional peptide, adrenomedullin. Int. Rev. Cytol..

[13] Martínez-Herrero S., Pérez-Matute P., Villanueva-Millán M., Oteo J., Martínez A. Changes in Gut Microbiota Induced by Lack of Adrenomedullin. Microbiology ASF. Proceedings of the 54th Interscience Conference on Antimicrobial Agents and Chemotherapy (ICAAC®).

[14] Martinez-Herrero S., Larrayoz I.M., Narro-Iniguez J., Villanueva-Millan M.J., Recio-Fernandez E., Perez-Matute P., Oteo J.A., Martínez A. (2016). Lack of Adrenomedullin Results in Microbiota Changes and Aggravates Azoxymethane and Dextran Sulfate Sodium-Induced Colitis in Mice. Front. Physiol..

[15] Martinez-Herrero S., Larrayoz I.M., Narro-Iniguez J., Rubio-Mediavilla S., Martinez A. (2017). Lack of Adrenomedullin Aggravates Acute TNBS-Induced Colitis Symptoms in Mice, Especially in Females. Front. Physiol..

[16] Clementi G., Caruso A., Cutuli V.M., de Bernardis E., Prato A., Mangano N.G., Amico-Roxas M. (1998). Effects of centrally of peripherally injected adrenomedullin on reserpine-induced gastric lesions. Eur. J. Pharmacol..

[17] Ashizuka S., Inatsu H., Kita T., Kitamura K. (2016). Adrenomedullin Therapy in Patients with Refractory Ulcerative Colitis: A Case Series. Dig. Dis. Sci..

[18] Ashizuka S., Kuroishi N., Nakashima K., Inatsu H., Kita T., Kitamura K. (2019). Adrenomedullin: A Novel Therapy for Intractable Crohn’s Disease with a Loss of Response to Infliximab. Intern. Med..

[19] Ashizuka S., Kita T., Inatsu H., Kitamura K. (2021). Adrenomedullin: A Novel Therapeutic for the Treatment of Inflammatory Bowel Disease. Biomedicines.

[20] Kitamura K., Kangawa K., Kawamoto M., Ichiki Y., Nakamura S., Matsuo H., Eto T. (1993). Adrenomedullin: A novel hypotensive peptide isolated from human pheochromocytoma. Biochem. Biophys. Res. Commun..

[21] Ishimitsu T., Kojima M., Kangawa K., Hino J., Matsuoka H., Kitamura K., Eto T., Matsuo H. (1994). Genomic structure of human adrenomedullin gene. Biochem. Biophys. Res. Commun..

[22] Beltowski J., Jamroz A. (2004). Adrenomedullin—What do we know 10 years since its discovery?. Pol. J. Pharmacol..

[23] Martínez A., Hodge D.L., Garayoa M., Young H.A., Cuttitta F. (2001). Alternative splicing of the proadrenomedullin gene results in differential expression of gene products. J. Mol. Endocrinol..

[24] Takei Y., Inoue K., Ogoshi M., Kawahara T., Bannai H., Miyano S. (2004). Identification of novel adrenomedullin in mammals: A potent cardiovascular and renal regulator. FEBS Lett..

[25] Roh J., Chang C.L., Bhalla A., Klein C., Hsu S.Y. (2004). Intermedin is a calcitonin/calcitonin gene-related peptide family peptide acting through the calcitonin receptor-like receptor/receptor activity-modifying protein receptor complexes. J. Biol. Chem..

[26] Perez-Castells J., Martin-Santamaria S., Nieto L., Ramos A., Martinez A., de Pascual-Teresa B., Jimenez-Barbero J. (2012). Structure of Micelle-Bound Adrenomedullin: A First Step Toward the Analysis of Its Interactions with Receptors and Small Molecules. Biopolymers.

[27] Lucyk S., Taha H., Yamamoto H., Miskolzie M., Kotovych G. (2006). NMR conformational analysis of proadrenomedullin N-terminal 20 peptide, a proangiogenic factor involved in tumor growth. Biopolymers.

[28] Martinez A., Bengoechea J.A., Cuttitta F. (2006). Molecular evolution of proadrenomedullin N-terminal 20 peptide (PAMP): Evidence for gene co-option. Endocrinology.

[29] Garayoa M., Martínez A., Lee S., Pío R., An W.G., Neckers L., Trepel J., Montuenga L.M., Ryan H., Johnson R. (2000). Hypoxia-inducible factor-1 (HIF-1) up-regulates adrenomedullin expression in human tumor cell lines during oxygen deprivation: A possible promotion mechanism of carcinogenesis. Mol. Endocrinol..

[30] Poyner D.R., Sexton P.M., Marshall I., Smith D.M., Quirion R., Born W., Muff R., Fischer J.A., Foord S.M. (2002). International Union of Pharmacology. XXXII. The mammalian calcitonin gene-related peptides, adrenomedullin, amylin, and calcitonin receptors. Pharmacol. Rev..

[31] Qi T., Christopoulos G., Bailey R.J., Christopoulos A., Sexton P.M., Hay D.L. (2008). Identification of N-terminal receptor activity-modifying protein residues important for calcitonin gene-related peptide, adrenomedullin, and amylin receptor function. Mol. Pharmacol..

[32] Gibbons C., Dackor R., Dunworth W., Fritz-Six K., Caron K.M. (2007). Receptor activity-modifying proteins: RAMPing up adrenomedullin signaling. Mol. Endocrinol..

[33] Kamohara M., Matsuo A., Takasaki J., Kohda M., Matsumoto M., Matsumoto S., Soga T., Hiyama H., Katou M. (2005). Identification of MrgX2 as a human G-protein-coupled receptor for proadrenomedullin N-terminal peptides. Biochem. Biophys. Res. Commun..

[34] Larrayoz I.M., Martinez-Herrero S., Ochoa-Callejero L., Garcia-Sanmartin J., Martinez A. (2013). Is the Cytoskeleton an Intracellular Receptor for Adrenomedullin and PAMP?. Curr. Protein Pept. Sci..

[35] Meyrath M., Palmer C.B., Reynders N., Vanderplasschen A., Ollert M., Bouvier M., Szpakowska M., Chevigné A. (2021). Proadrenomedullin N-Terminal 20 Peptides (PAMPs) Are Agonists of the Chemokine Scavenger Receptor ACKR3/CXCR7. ACS Pharmacol. Transl. Sci..

[36] Bachelerie F., Ben-Baruch A., Burkhardt A.M., Combadiere C., Farber J.M., Graham G.J., Horuk R., Sparre-Ulrich A.H., Locati M., Luster A.D. (2014). International Union of Basic and Clinical Pharmacology. [corrected]. LXXXIX. Update on the extended family of chemokine receptors and introducing a new nomenclature for atypical chemokine receptors. Pharmacol. Rev..

[37] Regard J.B., Sato I.T., Coughlin S.R. (2008). Anatomical profiling of G protein-coupled receptor expression. Cell.

[38] Garayoa M., Bodegas E., Cuttitta F., Montuenga L.M. (2002). Adrenomedullin in mammalian embryogenesis. Microsc. Res. Tech..

[39] Hayashi K.G., Hosoe M., Sakumoto R., Takahashi T. (2013). Temporo-spatial expression of adrenomedullin and its receptors in the bovine placenta. Reprod. Biol. Endocrinol..

[40] Shindo T., Kurihara Y., Nishimatsu H., Moriyama N., Kakoki M., Wang Y., Imai Y., Ebihara A., Kuwaki T., Ju K.-H. (2001). Vascular abnormalities and elevated blood pressure in mice lacking adrenomedullin gene. Circulation.

[41] Shimosawa T., Shibagaki Y., Ishibashi K., Kitamura K., Kangawa K., Kato S., Ando K., Fujita T. (2002). Adrenomedullin, an endogenous peptide, counteracts cardiovascular damage. Circulation.

[42] Dackor R.T., Fritz-Six K., Dunworth W.P., Gibbons C.L., Smithies O., Caron K.M. (2006). Hydrops fetalis, cardiovascular defects, and embryonic lethality in mice lacking the calcitonin receptor-like receptor gene. Mol. Cell Biol..

[43] Ichikawa-Shindo Y., Sakurai T., Kamiyoshi A., Kawate H., Iinuma N., Yoshizawa T., Koyama T., Fukuchi J., Iimuro S., Moriyama N. (2008). The GPCR modulator protein RAMP2 is essential for angiogenesis and vascular integrity. J. Clin. Investig..

[44] Dackor R., Fritz-Six K., Smithies O., Caron K. (2007). Receptor activity-modifying proteins 2 and 3 have distinct physiological functions from embryogenesis to old age. J. Biol. Chem..

[45] Plück A. (1996). Conditional mutagenesis in mice: The Cre/loxP recombination system. Int. J. Exp. Pathol..

[46] Nicholls M.G. (2004). Hemodynamic and hormonal actions of adrenomedullin. Braz. J. Med. Biol. Res..

[47] Samson W.K., Murphy T.C., Resch Z.T. (1998). Central mechanisms for the hypertensive effects of preproadrenomedullin-derived peptides in conscious rats. Am. J. Physiol..

[48] Martinez A. (2006). A new family of angiogenic factors. Cancer Lett..

[49] Koyama T., Ochoa-Callejero L., Sakurai T., Kamiyoshi A., Ichikawa-Shindo Y., Iinuma N., Arai T., Yoshizawa T., Iesato Y., Lei Y. (2013). Vascular Endothelial Adrenomedullin-RAMP2 System Is Essential for Vascular Integrity and Organ Homeostasis. Circulation.

[50] Garcia-Honduvilla N., Cifuentes A., Bellon J.M., Bujan J., Martinez A. (2013). The angiogenesis promoter, proadrenomedullin N-terminal 20 peptide (PAMP), improves healing in both normoxic and ischemic wounds either alone or in combination with autologous stem/progenitor cells. Histol. Histopathol..

[51] Harada K., Yamahara K., Ohnishi S., Otani K., Kanoh H., Ishibashi-Ueda H., Minamino N., Kangawa K., Nagaya N., Ikeda T. (2011). Sustained-release adrenomedullin ointment accelerates wound healing of pressure ulcers. Regul. Pept..

[52] Nishikimi T. (2007). Adrenomedullin in the kidney-renal physiological and pathophysiological roles. Curr. Med. Chem..

[53] Jougasaki M., Wei C.M., Aarhus L.L., Heublein D.M., Sandberg S.M., Burnett J.C. (1995). Renal localization and actions of adrenomedullin: A natriuretic peptide. Am. J. Physiol..

[54] López J., Cuesta N., Martínez A., Montuenga L., Cuttitta F. (1999). Proadrenomedullin N-terminal 20 peptide (PAMP) immunoreactivity in vertebrate juxtaglomerular granular cells identified by both light and electron microscopy. Gen. Comp. Endocrinol..

[55] Zudaire E., Cuttitta F., Martínez A. (2003). Regulation of pancreatic physiology by adrenomedullin and its binding protein. Regul. Pept..

[56] Martinez-Herrero S., Larrayoz I.M., Ochoa-Callejero L., Fernandez L.J., Allueva A., Ochoa I., Martínez A. (2016). Prevention of Bone Loss in a Model of Postmenopausal Osteoporosis through Adrenomedullin Inhibition. Front. Physiol..

[57] Goldstein J.L., Zhao T.J., Li R.L., Sherbet D.P., Liang G., Brown M.S. (2011). Surviving starvation: Essential role of the ghrelin-growth hormone axis. Cold Spring Harb. Symp. Quant. Biol..

[58] Serrano J., Alonso D., Fernández A.P., Encinas J.M., López J.C., Castro-Blanco S., Fernández-Vizarra P., Richart A., Santacana M., Uttenthal L.O. (2002). Adrenomedullin in the central nervous system. Microsc. Res. Tech..

[59] Miyashita K., Itoh H., Arai H., Suganami T., Sawada N., Fukunaga Y., Sone M., Yamahara K., Yurugi-Kobayashi T., Park K. (2006). The neuroprotective and vasculo-neuro-regenerative roles of adrenomedullin in ischemic brain and its therapeutic potential. Endocrinology.

[60] Saita M., Shimokawa A., Kunitake T., Kato K., Hanamori T., Kitamura K., Eto T., Kannan H. (1998). Central actions of adrenomedullin on cardiovascular parameters and sympathetic outflow in conscious rats. Am. J. Physiol..

[61] Kis B., Abrahám C.S., Deli M.A., Kobayashi H., Niwa M., Yamashita H., Busija D.W., Ueta Y. (2003). Adrenomedullin, an autocrine mediator of blood-brain barrier function. Hypertens Res..

[62] Fernandez A.P., Serrano J., Tessarollo L., Cuttitta F., Martinez A. (2008). Lack of adrenomedullin in the mouse brain results in behavioral changes, anxiety, and lower survival under stress conditions. Proc. Natl. Acad. Sci. USA.

[63] Fernandez A.P., Serrano J., Martinez-Murillo R., Martinez A. (2010). Lack of Adrenomedullin in the Central Nervous System Results in Apparently Paradoxical Alterations on Pain Sensitivity. Endocrinology.

[64] Gröschl M., Wendler O., Topf H.G., Bohlender J., Köhler H. (2009). Significance of salivary adrenomedullin in the maintenance of oral health: Stimulation of oral cell proliferation and antibacterial properties. Regul. Pept..

[65] Hiroshima Y., Bando M., Kataoka M., Inagaki Y., Herzberg M.C., Ross K.F., Hosoi K., Nagata T., Kido J.-I. (2011). Regulation of antimicrobial peptide expression in human gingival keratinocytes by interleukin-1α. Arch. Oral. Biol..

[66] McLachlan J.L., Smith A.J., Bujalska I.J., Cooper P.R. (2005). Gene expression profiling of pulpal tissue reveals the molecular complexity of dental caries. Biochim. Biophys. Acta.

[67] Wang H., Tomikawa M., Jones M.K., Sarfeh I.J., Tarnawski A.S. (1999). Ethanol injury triggers activation of adrenomedullin and its receptor genes in gastric mucosa. Dig. Dis. Sci..

[68] Fukuda K., Tsukada H., Oya M., Onomura M., Kodama M., Nakamura H., Hosokawa M., Seino Y. (1999). Adrenomedullin promotes epithelial restitution of rat and human gastric mucosa in vitro. Peptides.

[69] Sakata J., Asada Y., Shimokubo T., Kitani M., Inatsu H., Kitamura K., Kangawa K., Matsuo H., Sumiyoshi A., Eto T. (1998). Adrenomedullin in the gastrointestinal tract. Distribution and gene expression in rat and augmented gastric adrenomedullin after fasting. J. Gastroenterol..

[70] Rossowski W.J., Jiang N.Y., Coy D.H. (1997). Adrenomedullin, amylin, calcitonin gene-related peptide and their fragments are potent inhibitors of gastric acid secretion in rats. Eur. J. Pharmacol..

[71] Rossowski W.J., Cheng B.L., Jiang N.Y., Coy D.H. (1998). Examination of somatostatin involvement in the inhibitory action of GIP, GLP-1, amylin and adrenomedullin on gastric acid release using a new SRIF antagonist analogue. Br. J. Pharmacol..

[72] Hirsch A.B., McCuen R.W., Arimura A., Schubert M.L. (2003). Adrenomedullin stimulates somatostatin and thus inhibits histamine and acid secretion in the fundus of the stomach. Regul. Pept..

[73] Clementi G., Caruso A., Cutuli V.M., Mangano N.G., Salomone S., Lempereur L., Prato A., Matera M., Amico-Roxas M. (2002). Gastroprotective effect of adrenomedullin administered subcutaneously in the rat. Peptides.

[74] Salomone S., Caruso A., Cutuli V.M., Mangano N.G., Prato A., Amico-Roxas M., Bianchi A., Clementi G. (2003). Effects of adrenomedullin on the contraction of gastric arteries during reserpine-induced gastric ulcer. Peptides.

[75] Martínez V., Cuttitta F., Taché Y. (1997). Central action of adrenomedullin to inhibit gastric emptying in rats. Endocrinology.

[76] Holzer-Petsche U., Seitz H., Lembeck F. (1989). Effect of capsaicin on gastric corpus smooth muscle of the rat in vitro. Eur. J. Pharmacol..

[77] Ebert E.C. (2010). The thyroid and the gut. J. Clin. Gastroenterol..

[78] Mulder H., Ahren B., Karlsson S., Sundler F. (1996). Adrenomedullin: Localization in the gastrointestinal tract and effects on insulin secretion. Regul. Pept..

[79] Hussain S., Miyazawa R., Tomomasa T., Kaneko H., Takahashi A., Watanabe T., Arakawa H., Morikawa A. (2005). Possible involvement of adrenomedullin in lipopolysaccharide-induced small-intestinal motility changes in conscious rats. J. Gastroenterol..

[80] Zhou M., Chaudry I.H., Wang P. (2001). The small intestine is an important source of adrenomedullin release during polymicrobial sepsis. Am. J. Physiol. Regul. Integr. Comp. Physiol..

[81] Fukuda K., Tsukada H., Onomura M., Saito T., Kodama M., Nakamura H., Taniguchi T., Tominaga M., Hosokawa M., Seino Y. (1998). Effect of adrenomedullin on ion transport and muscle contraction in rat distal colon. Peptides.

[82] Kiyomizu A., Kitamura K., Kawamoto M., Eto T. (2001). Distribution and molecular forms of adrenomedullin and proadrenomedullin N-terminal 20 peptide in the porcine gastrointestinal tract. J. Gastroenterol..

[83] Kravtsov G.M., Hwang I.S., Tang F. (2004). The inhibitory effect of adrenomedullin in the rat ileum: Cross-talk with beta3-adrenoceptor in the serotonin-induced muscle contraction. J. Pharmacol. Exp. Ther..

[84] Takahashi T. (2013). Interdigestive migrating motor complex -its mechanism and clinical importance. J. Smooth Muscle Res..

[85] Fernández de Arcaya I., Lostao M.P., Martínez A., Berjón A., Barber A. (2005). Effect of adrenomedullin and proadrenomedullin N-terminal 20 peptide on sugar transport in the rat intestine. Regul. Pept..

[86] Martínez A., Cuttitta F., Teitelman G. (1998). Expression pattern for adrenomedullin during pancreatic development in the rat reveals a common precursor with other endocrine cell types. Cell Tissue Res..

[87] Martínez A., Weaver C., López J., Bhathena S.J., Elsasser T.H., Miller M.J., Moody T.W., Unsworth E.J., Cuttitta F. (1996). Regulation of insulin secretion and blood glucose metabolism by adrenomedullin. Endocrinology.

[88] Sekine N., Takano K., Kimata-Hayashi N., Kadowaki T., Fujita T. (2006). Adrenomedullin inhibits insulin exocytosis via pertussis toxin-sensitive G protein-coupled mechanism. Am. J. Physiol. Endocrinol. Metab..

[89] Martínez A., Pío R., López J., Cuttitta F. (2001). Expression of the adrenomedullin binding protein, complement factor H, in the pancreas and its physiological impact on insulin secretion. J. Endocrinol..

[90] Tsuchida T., Ohnishi H., Tanaka Y., Mine T., Fujita T. (1999). Inhibition of stimulated amylase secretion by adrenomedullin in rat pancreatic acini. Endocrinology.

[91] Wan X., Song M., Wang A., Zhao Y., Wei Z., Lu Y. (2021). Microbiome Crosstalk in Immunotherapy and Antiangiogenesis Therapy. Front. Immunol..

[92] Trakman G.L., Fehily S., Basnayake C., Hamilton A.L., Russell E., Wilson-O’Brien A., Kamm M.A. (2021). Diet and gut microbiome in gastrointestinal disease. J. Gastroenterol. Hepatol..

[93] Martínez A., Elsasser T.H., Muro-Cacho C., Moody T.W., Miller M.J., Macri C.J., Cuttitta F. (1997). Expression of adrenomedullin and its receptor in normal and malignant human skin: A potential pluripotent role in the integument. Endocrinology.

[94] Allaker R.P., Kapas S. (2003). Adrenomedullin and mucosal defence: Interaction between host and microorganism. Regul. Pept..

[95] Kishikawa H., Nishida J., Ichikawa H., Kaida S., Morishita T., Miura S., Hibi T. (2009). Lipopolysaccharides stimulate adrenomedullin synthesis in intestinal epithelial cells: Release kinetics and secretion polarity. Peptides.

[96] Matheson P.J., Mays M.P., Hurt R.T., Harris P.D., Garrison R.N. (2003). Adrenornedullin is increased in the portal circulation during chronic sepsis in rats. Am. J. Surg..

[97] Walsh T.J., Martinez A., Peter J., Unsworth E., Cuttitta F. (1996). Antimicrobial activity of adrenomedullin and its gene-related peptides. Clin. Infect. Dis..

[98] Allaker R.P., Zihni C., Kapas S. (1999). An investigation into the antimicrobial effects of adrenomedullin on members of the skin, oral, respiratory tract and gut microflora. FEMS Immunol. Med. Microbiol..

[99] Kapas S., Bansal A., Bhargava V., Maher R., Malli D., Hagi-Pavli E., Allaker R.P. (2001). Adrenomedullin expression in pathogen-challenged oral epithelial cells. Peptides.

[100] Marutsuka K., Nawa Y., Asada Y., Hara S., Kitamura K., Eto T., Sumiyoshi A. (2001). Adrenomedullin and proadrenomudullin N-terminal 20 peptide (PAMP) are present in human colonic epithelia and exert an antimicrobial effect. Exp. Physiol..

[101] Allaker R.P., Grosvenor P.W., McAnerney D.C., Sheehan B.E., Srikanta B.H., Pell K., Kapas S. (2006). Mechanisms of adrenomedullin antimicrobial action. Peptides.

[102] Azhari H., Underwood F., King J., Coward S., Shah S., Ng S., Ho G., Chan C., Tang W., Kaplan G.G. (2018). The Global Incidence of Peptic Ulcer Disease and Its Complications at the Turn of the 21st Century: A Systematic Review. Am. J. Gastroenterol..

[103] Kaneko H., Mitsuma T., Nagai H., Mori S., Iyo T., Kusugami K., Tache Y. (1998). Central action of adrenomedullin to prevent ethanol-induced gastric injury through vagal pathways in rats. Am. J. Physiol..

[104] Egerod K.L., Engelstoft M.S., Lund M.L., Grunddal K.V., Zhao M., Barir-Jensen D., Nygaard E.B., Petersen N., Holst J.J., Schwartz T.W. (2015). Transcriptional and Functional Characterization of the G Protein-Coupled Receptor Repertoire of Gastric Somatostatin Cells. Endocrinology.

[105] Khadzhiev O.C., Lupal’tsov V.I., Simonenkov A.P., Klimenko N.A., Tatarko S.V. (2000). Microcirculatory disturbances in gastric mucosa during ulcer disease and effects of serotonin on their dynamics. Bull. Exp. Biol. Med..

[106] Hashimoto H., Akimoto M., Maeda A., Shigemoto M., Yamashita K., Yokoyama I. (2000). Changes in vasoactive substances during gastric ulcer healing. J. Cardiovasc. Pharmacol..

[107] Cudnik M.T., Darbha S., Jones J., Macedo J., Stockton S.W., Hiestand B.C. (2013). The diagnosis of acute mesenteric ischemia: A systematic review and meta-analysis. Acad. Emerg. Med..

[108] Coelho A., Logo M., Gouveia R., Campos J., Augusto R., Canedo A. (2016). Acute Mesenteric Ischemia: Epidemiology, Risk Ractors and Determinants of Mortality. Rev. Port. Cir. Cardiotorac. Vasc..

[109] Oldenburg W.A., Lau L.L., Rodenberg T.J., Edmonds H.J., Burger C.D. (2004). Acute mesenteric ischemia: A clinical review. Arch. Intern. Med..

[110] Higuchi S., Wu R., Zhou M., Marini C.P., Ravikumar T.S., Wang P. (2008). Gut Hyperpermiability after Ischemia and Reperfusion: Attenuation with Adrenomedullin and its Binding Protein Treatment. Int. J. Clin. Exp. Pathol..

[111] Zhang F., Wu R., Zhou M., Blau S.A., Wang P. (2009). Human adrenomedullin combined with human adrenomedullin binding protein-1 is protective in gut ischemia and reperfusion injury in the rat. Regul. Pept..

[112] Looi Y.H., Kane K.A., McPhaden A.R., Wainwright C.L. (2006). Adrenomedullin acts via nitric oxide and peroxynitrite to protect against myocardial ischaemia-induced arrhythmias in anaesthetized rats. Br. J. Pharmacol..

[113] Okumura H., Nagaya N., Itoh T., Okano I., Hino J., Mori K., Tsukamoto Y., Ishibashi-Ueda H., Miwa S., Tambara K. (2004). Adrenomedullin infusion attenuates myocardial ischemia/reperfusion injury through the phosphatidylinositol 3-kinase/Akt-dependent pathway. Circulation.

[114] Nishimatsu H., Hirata Y., Shindo T., Kurihara H., Kakoki M., Nagata D., Hayakawa H., Satonaka H., Sata M., Tojo A. (2002). Role of endogenous adrenomedullin in the regulation of vascular tone and ischemic renal injury: Studies on transgenic/knockout mice of adrenomedullin gene. Circ. Res..

[115] Wong H.K., Tang F., Cheung T.T., Cheung B.M. (2014). Adrenomedullin and diabetes. World J. Diabetes.

[116] Rulle S., Ah Kioon M.D., Asensio C., Mussard J., Ea H.K., Boissier M.C., Lioté F., Falgarone G. (2012). Adrenomedullin, a neuropeptide with immunoregulatory properties induces semi-mature tolerogenic dendritic cells. Immunology.

[117] García-Unzueta M.T., Montalbán C., Pesquera C., Berrazueta J.R., Amado J.A. (1998). Plasma adrenomedullin levels in type 1 diabetes. Relationship with clinical parameters. Diabetes Care.

[118] Martínez A., Elsasser T.H., Bhathena S.J., Pío R., Buchanan T.A., Macri C.J., Cuttitta F. (1999). Is adrenomedullin a causal agent in some cases of type 2 diabetes?. Peptides.

[119] Larrayoz I.M., Martinez-Herrero S., Garcia-Sanmartin J., Ochoa-Callejero L., Martinez A. (2014). Adrenomedullin and tumour microenvironment. J. Transl. Med..

[120] Sion-Vardy N., Tzikinovsky A., Bolotyn A., Segal S., Fishman D. (2004). Augmented expression of chromogranin A and serotonin in peri-malignant benign prostate epithelium as compared to adenocarcinoma. Pathol. Res. Pract..

[121] Uemura M., Yamamoto H., Takemasa I., Mimori K., Mizushima T., Ikeda M., Sekimoto M., Doki Y., Mori M. (2011). Hypoxia-inducible adrenomedullin in colorectal cancer. Anticancer Res..

[122] Benyahia Z., Dussault N., Cayol M., Sigaud R., Berenguer-Daizé C., Delfino C., Tounsi A., Garcia S., Martin P.M., Mabrouk K. (2017). Stromal fibroblasts present in breast carcinomas promote tumor growth and angiogenesis through adrenomedullin secretion. Oncotarget.

[123] Baranello C., Mariani M., Andreoli M., Fanelli M., Martinelli E., Ferrandina G., Scambia G., Shahabi S., Ferlini C. (2012). Adrenomedullin in ovarian cancer: Foe in vitro and friend in vivo?. PLoS ONE.

[124] Keleg S., Kayed H., Jiang X., Penzel R., Giese T., Büchler M.W., Friess H., Kleeff J. (2007). Adrenomedullin is induced by hypoxia and enhances pancreatic cancer cell invasion. Int. J. Cancer.

[125] Letizia C., Tamburrano G., Alo P., Paoloni A., Caliumi C., Marinoni E., di Iorio R., d’Erasmo E. (2001). Adrenomedullin, a new peptide, in patients with insulinoma. Eur. J. Endocrinol..

[126] Aggarwal G., Ramachandran V., Javeed N., Arumugam T., Dutta S., Klee G.G., Smyrk T.C., Bamlet W., Han J.J., Vittar N.B.R. (2012). Adrenomedullin is up-regulated in patients with pancreatic cancer and causes insulin resistance in β cells and mice. Gastroenterology.

[127] Xu M., Qi F., Zhang S., Ma X., Wang S., Wang C., Fu Y., Luo Y. (2016). Adrenomedullin promotes the growth of pancreatic ductal adenocarcinoma through recruitment of myelomonocytic cells. Oncotarget.

[128] Qiao F., Fang J., Xu J., Zhao W., Ni Y., Akuo B.A., Zhang W., Liu Y., Ding F., Li G. (2017). The role of adrenomedullin in the pathogenesis of gastric cancer. Oncotarget.

[129] Nouguerede E., Berenguer C., Garcia S., Bennani B., Delfino C., Nanni I., Dahan L., Gasmi M., Seitz J.-F., Martin P.-M. (2013). Expression of adrenomedullin in human colorectal tumors and its role in cell growth and invasion in vitro and in xenograft growth in vivo. Cancer Med..

[130] Ochoa-Callejero L., Garcia-Sanmartin J., Martinez-Herrero S., Rubio-Mediavilla S., Narro-Iniguez J., Martinez A. (2017). Small molecules related to adrenomedullin reduce tumor burden in a mouse model of colitis-associated colon cancer. Sci. Rep..

[131] Leone V., Chang E.B., Devkota S. (2013). Diet, microbes, and host genetics: The perfect storm in inflammatory bowel diseases. J. Gastroenterol..

[132] Ford A.C., Moayyedi P., Hanauer S.B. (2013). Ulcerative colitis. BMJ.

[133] Jergens A.E., Simpson K.W. (2012). Inflammatory bowel disease in veterinary medicine. Front. Biosci..

[134] Yesudhas D., Gosu V., Anwar M.A., Choi S. (2014). Multiple roles of toll-like receptor 4 in colorectal cancer. Front. Immunol..

[135] Ha C.W., Lam Y.Y., Holmes A.J. (2014). Mechanistic links between gut microbial community dynamics, microbial functions and metabolic health. World J. Gastroenterol..

[136] Pedreño M., Morell M., Robledo G., Souza-Moreira L., Forte-Lago I., Caro M., O’Valle F., Ganea D., Gonzalez-Rey E. (2014). Adrenomedullin protects from experimental autoimmune encephalomyelitis at multiple levels. Brain Behav. Immun..

[137] Gonzalez-Rey E., Delgado-Maroto V., Souza Moreira L., Delgado M. (2010). Neuropeptides as therapeutic approach to autoimmune diseases. Curr. Pharm. Des..

[138] Ashizuka S., Ishikawa N., Kato J., Yamaga J., Inatsu H., Eto T., Kitamura K. (2005). Effect of adrenomedullin administration on acetic acid-induced colitis in rats. Peptides.

[139] Ashizuka S., Inagaki-Ohara K., Kuwasako K., Kato J., Inatsu H., Kitamura K. (2009). Adrenomedullin treatment reduces intestinal inflammation and maintains epithelial barrier function in mice administered dextran sulphate sodium. Microbiol. Immunol..

[140] Talero E., Sanchez-Fidalgo S., de la Lastra C.A., Illanes M., Calvo J.R., Motilva V. (2008). Acute and chronic responses associated with adrenomedullin administration in experimental colitis. Peptides.

[141] MacManus C.F., Campbell E.L., Keely S., Burgess A., Kominsky D.J., Colgan S.P. (2011). Anti-inflammatory actions of adrenomedullin through fine tuning of HIF stabilization. FASEB J..

[142] Kinoshita Y., Arita S., Murazoe H., Kitamura K., Ashizuka S., Inagaki-Ohara K. (2019). Subcutaneously administered adrenomedullin exerts a potent therapeutic effect in a murine model of ulcerative colitis. Hum. Cell..

[143] Yi Z., Fan H., Liu X., Tang Q., Zuo D., Yang J. (2015). Adrenomedullin improves intestinal epithelial barrier function by downregulating myosin light chain phosphorylation in ulcerative colitis rats. Mol. Med. Rep..

[144] Hayashi Y., Narumi K., Tsuji S., Tsubokawa T., Nakaya M.A., Wakayama T., Zuka M., Ohshima T., Yamagishi M., Okada T. (2011). Impact of adrenomedullin on dextran sulfate sodium-induced inflammatory colitis in mice: Insights from in vitro and in vivo experimental studies. Int. J. Colorectal. Dis..

[145] Hayakawa H., Hirata Y., Kakoki M., Suzuki Y., Nishimatsu H., Nagata D., Suzuki E., Kikuchi K., Nagano T., Kangawa K. (1999). Role of nitric oxide-cGMP pathway in adrenomedullin-induced vasodilation in the rat. Hypertension.

[146] Talero E., Sanchez-Fidalgo S., Villegas I., de la Lastra C.A., Illanes M., Motilva V. (2011). Role of different inflammatory and tumor biomarkers in the development of ulcerative colitis-associated carcinogenesis. Inflamm. Bowel Dis..

[147] Davis R.B., Kechele D.O., Blakeney E.S., Pawlak J.B., Caron K.M. (2017). Lymphatic deletion of calcitonin receptor-like receptor exacerbates intestinal inflammation. JCI Insight.

[148] Zudaire E., Portal-Núñez S., Cuttitta F. (2006). The central role of adrenomedullin in host defense. J. Leukoc. Biol..

[149] Ashizuka S., Inatsu H., Inagaki-Ohara K., Kita T., Kitamura K. (2013). Adrenomedullin as a Potential Therapeutic Agent for Inflammatory Bowel Disease. Curr. Protein Pept. Sci..

[150] Liang J., Sha S.M., Wu K.C. (2014). Role of the intestinal microbiota and fecal transplantation in inflammatory bowel diseases. J. Dig. Dis..

[151] Ashizuka S., Inatsu H., Kita T., Kitamura K. (2012). The First Clinical Pilot Study of Adrenomedullin Therapy in Refractory Ulcerative Colitis: The Initial Six Cases. Gastroenterology.

[152] Ashizuka S., Kita T., Inatsu H., Kitamura K. (2013). Adrenomedullin: A Novel Therapy for Intractable Ulcerative Colitis. Inflamm. Bowel Dis..

[153] Lainchbury J.G., Troughton R.W., Lewis L.K., Yandle T.G., Richards A.M., Nicholls M.G. (2000). Hemodynamic, hormonal, and renal effects of short-term adrenomedullin infusion in healthy volunteers. J. Clin. Endocrinol. Metab..

[154] Nagaya N., Goto Y., Satoh T., Sumida H., Kojima S., Miyatake K., Kangawa K. (2002). Intravenous adrenomedullin in myocardial function and energy metabolism in patients after myocardial infarction. J. Cardiovas. Pharmacol..

[155] Troughton R.W., Lewis L.K., Yandle T.G., Richards A.M., Nicholls M.G. (2000). Hemodynamic, hormone, and urinary effects of adrenomedullin infusion in essential hypertension. Hypertension.

[156] Kataoka Y., Miyazaki S., Yasuda S., Nagaya N., Noguchi T., Yamada N., Morii I., Kawamura A., Doi K., Miyatake K. (2010). The First Clinical Pilot Study of Intravenous Adrenomedullin Administration in Patients With Acute Myocardial Infarction. J. Cardiovasc. Pharmacol..

[157] Nagata S., Yamasaki M., Kitamura K. (2017). Anti-Inflammatory Effects of PEGylated Human Adrenomedullin in a Mouse DSS-Induced Colitis Model. Drug Dev. Res..

[158] Martinez A., Julian M., Bregonzio C., Notari L., Moody T.W., Cuttitta F. (2004). Identification of vasoactive nonpeptidic positive and negative modulators of adrenomedullin using a neutralizing antibody-based screening strategy. Endocrinology.

[159] Kong H., You N., Chen H., Teng Y.S., Liu Y.G., Lv Y.P., Mao F.Y., Cheng P., Chen W., Zhao Z. (2020). Helicobacter pylori-induced adrenomedullin modulates IFN-γ-producing T-cell responses and contributes to gastritis. Cell Death Dis..

[160] Kim J.Y., Park W.D., Lee S., Park J.H. (2012). Adrenomedullin is involved in the progression of colonic adenocarcinoma. Mol. Med. Rep..

[161] Kaafarani I., Fernandez-Sauze S., Berenguer C., Chinot O., Delfino C., Dussert C., Metellus P., Boudouresque F., Mabrouk K., Grisoli F. (2009). Targeting adrenomedullin receptors with systemic delivery of neutralizing antibodies inhibits tumor angiogenesis and suppresses growth of human tumor xenografts in mice. FASEB J..

